# On the recovery of disorders of consciousness under intrathecal baclofen administration for severe spasticity—An observational study

**DOI:** 10.1002/brb3.2566

**Published:** 2022-04-10

**Authors:** Lucas‐Michael Halbmayer, Markus Kofler, Gabriel Hitzenberger, Heinrich Matzak, Elena Fava, Eleonora Genelin, Mario Werkmann, Leopold Saltuari, Viviana Versace, Judith Dobesberger, Elke Pucks‐Faes

**Affiliations:** ^1^ Department of Neurology Hochzirl Hospital Zirl Austria; ^2^ Department of Neurorehabilitation Hospital of Vipiteno (SABES‐ASDAA) Vipiteno Italy; ^3^ Research Unit for Neurorehabilitation Bolzano Italy; ^4^ Rehabilitation Center Großgmain, Pensionsversicherungsanstalt Teaching Hospital of Paracelsus Medical University Salzburg Austria

**Keywords:** brainstem, Coma Recovery Scale‐Revised, consciousness recovery, disorders of consciousness, intrathecal baclofen, pedunculopontine nucleus, prepulse inhibition

## Abstract

**Background:**

Occasionally, patients show dramatic recovery from disorders of consciousness (DOC) under intrathecal baclofen (ITB), an established treatment option for severe supraspinal spasticity. Anecdotal explanations for ITB‐related recovery of cognition include modulation of afferent impulses at the spinal level, thereby reducing spasticity‐related proprioceptive information overload within cortico–thalamo–cortical connections.

**Objective:**

In this retrospective patient chart analysis, we assessed whether a reduction in spasticity would be associated with an increase in Coma Recovery Scale revised (CRS‐R) scores in a larger sample of patients than previously published.

**Methods:**

From a hospital‐based ITB treatment register, we extracted data from 26 patients with DOC and severe supraspinal spasticity who improved by >2 points on the Coma Recovery Scale revised (CRS‐R) within 6 months after ITB treatment initiation. We assessed Modified Ashworth scale (MAS) scores and CRS‐R scores on admission (PRE) and 3 and 6 months after initiation of ITB treatment (3M, 6M). We performed correlation analysis of the scores and their respective changes (PRE to 3M, 3M to 6M). We also correlated the time from acute event until ITB initiation to CRS‐R scores at 3M and 6M.

**Results:**

ITB led to significant improvement in spasticity based on MAS scores, which did not correlate to the improvements seen in CRS‐R total and subscale scores. Daily ITB dose did neither correlate to MAS scores nor to CRS‐total scores in the whole patient group, but after 3 months, ITB dose correlated to some CRS‐R subscale scores in some patient subgroups. Time until ITB treatment did not correlate to CRS‐R scores later on.

**Conclusions:**

Our data confirm that ITB may exert beneficial effects in selected DOC patients with respect to improved cognitive functions, which, however, do not correlate to its antispastic effect. The lack of correlation between time to ITB and CRS‐R outcome, but significant CRS‐R improvements following pump implantation, renders spontaneous remissions unlikely and leaves room for alternative pharmacological mechanisms.

## Graphical abstract

In this retrospective patient chart analysis, we assessed whether a reduction in spasticity would be associated with an increase in Coma Recovery Scale revised (CRS‐R) scores in a larger sample of patients than previously published. Our data confirm that ITB may exert beneficial effects in selected DOC patients with respect to improved cognitive functions, which, however, do not correlate to its antispastic effect. The lack of correlation between time to ITB and CRS‐R outcome, but significant CRS‐R improvements following pump implantation, renders spontaneous remissions unlikely and leaves room for alternative pharmacological mechanisms.

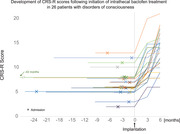

## 1 INTRODUCTION

Disorders of consciousness (DOC) following serious brain injury include coma, unresponsive wakefulness syndrome (UWS, previously referred to as vegetative state), and minimally conscious state (MCS) (Laureys et al., 2010). Although these disorders are clearly distinct, the recovery of consciousness is recognized as a clinical continuum that covers a wide range of consciousness gradations, ranging from coma to the restoration of full consciousness (Adams, 2000; The Multi‐Society Task Force on PVS, 1994a). The most common causes of DOC are vascular, traumatic, and anoxic brain injuries (Estraneo et al., 2021). The Coma Recovery Scale revised (CRS‐R) has been recommended as clinical diagnostic and prognostic tool to distinguish UWS from MCS; it explores auditory, visual, motor, oromotor/verbal, communication, and arousal processes (Giacino et al., 2004). Up to 89% of patients with disorders of consciousness develop moderate to severe spasticity, often resistant to oral medication (Thibaut et al., 2015). These patients are candidates for intrathecal baclofen (ITB) therapy to alleviate spasticity.

High‐dose systemic administration of baclofen, a selective GABA_B_ receptor agonist, is often accompanied by central nervous system side effects, such as reduced vigilance and respiratory or cardiovascular depression (Anand et al., 2017). Using an electronically programmable pump, baclofen can be delivered directly into the intrathecal space bypassing the blood–brain barrier, rendering therapeutic effects at very low doses (micrograms instead of milligrams), thus minimizing side effects. Since 1985, ITB has been an established treatment option for severe supraspinal spasticity (Albright, 1993; Becker et al., 1997; Dralle et al., 1985; Rawicki, 1999; Rifici et al., 1994; Saltuari et al., 1989). Often, the main objective of ITB treatment in patients with severe generalized spasticity is to improve comfort, and to enable better positioning and easier nursing care (Maneyapanda et al., 2017). Occasionally, patients experienced dramatic recovery from persistent vegetative state after initiation of ITB, which seems quite surprising as high doses of baclofen cause reduced vigilance, even coma (Kofler et al., 1992). Sporadically, improvements in vigilance or cognitive functions were briefly mentioned in the literature (Becker et al., 1997; François et al., 2001; Turner, 2003). To date, a total of 14 such patients have been reported in detail, typically in brief case descriptions (Al‐Khodairy et al., 2015; Formisano et al., 2019; Kawecki et al., 2007; Oyama et al., 2010; Prabin et al., 2011; Sarà et al., 2009; Taira, 2009).

Furthermore, two prospective studies covering different observation periods and applying different clinical scales analyzed the effect of ITB on awareness and cognitive abilities (Margetis et al., 2014; Posteraro et al., 2013). Posteraro et al. (2013) reported on 12 patients, that is, eight traumatic brain injuries (TBI), one cerebral hypoxia, three with cerebral hemorrhage, who received ITB within 6 months after the acute event. The authors assessed Modified Ashworth Scale (MAS), Spasm Frequency Score (Penn et al., 1989), Disability Rating Scale (DRS) Disability rating scale for severe head trauma: coma to community (Rappaport et al., 1982), and Level of Cognitive Functioning (LCF) (Malkmus & Booth, 1979) and observed significant improvements in all scores after 3 months (decrease in DRS in 11 patients, increase in LCF in 10 patients). Administered ITB doses ranged from 160 to 800 μg/day with a mean dosage of 380 μg/day. The authors found no difference in DRS and LCF in patients implanted within 3 months compared to those implanted within 6 months and recommended early ITB treatment initiation (Posteraro et al., 2013). Margetis et al. (2014) reported on eight patients with UWS and MCS (six TBI, one cerebral hypoxia, one hydrocephalus). ITB therapy was initiated 5–108 months after the acute event and daily ITB dose ranged from 50 to 800 μg. The authors noted improvement in CRS‐R scores in three patients. One improved by 1 point in the visual CRS‐R subscale score, 35 months following ITB treatment initiation and 9 years after TBI; one patient improved from CRS‐R 10 to 19, 3.5 years after initiating ITB treatment, with first signs of improvement appearing after 2.5 years corresponding to 5.5 years following TBI; and one patient improved from CRS‐R 11 to 22, into 2 years of ITB therapy, with first signs appearing 2.5 years after TBI (Margetis et al., 2014). These latter patients showed quite late progress after the beginning of ITB treatment; thus, it seems less evident that ITB was the key factor for their clinical improvement.

The mechanisms underlying ITB‐related improvement of awareness and cognition have so far only been hypothesized based on observation in only few patients. Pathophysiological theories put forward so far have suggested either a modulation of afferent impulses by ITB at the spinal level, thereby reducing a possible spasticity‐related proprioceptive information overload within the cortico–thalamo–cortical connections that might interfere with the maintenance of awareness, or a modulation of possibly dysregulated sleep–wake cycles (Sarà et al., 2009).

In this retrospective observational study, we sought to verify and confirm such an association of alleviated spasticity with the recovery of consciousness and improvement in cognitive functions in a larger patient sample than previously published. We were interested whether ITB might exert a beneficial effect in specific cognitive domains, and whether such an improvement could be attributed to reduced spasticity. If no such association were to be established, we would propose other alternative mechanisms being possibly involved in the recovery of cognition, which might inform future work to investigate such mechanisms.

## 2 PATIENTS AND METHODS

**FIGURE 1:**
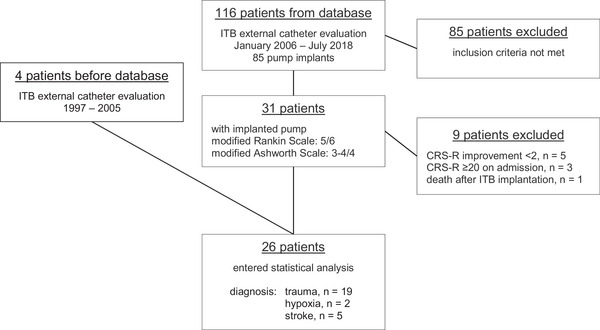
Flow chart

In this retrospective study, we selected 26 patients with a history of DOC from a hospital‐based ITB treatment register, who improved in CRS‐R scores within 6 months after ITB pump implantation. We intentionally included only patients who had gained at least 2 points in CRS‐R scores attempting to characterize this specific patient population. We compared data on spasticity and cognition before and 3 and 6 months after initiation of ITB treatment and correlated data with daily ITB dose. Between January 2006 and July 2018, 116 patients underwent continuous ITB testing with an external pump in the Department of Neurology at Hochzirl Hospital, Austria. After this standardized routine procedure in our institution to determine response magnitude and possible adverse effects (Pucks‐Faes et al., 2019), 85 patients underwent subsequent implantation of a permanent drug‐delivery system (Medtronic Synchromed II). Demographic and clinical characteristics are routinely collected in an ITB treatment register, which was used to identify DOC patients based on the following criteria on admission: diagnosis of TBI, cerebral hypoxia, intracranial hemorrhage, or subarachnoid hemorrhage; modified Rankin Scale of 5/6 (representing non‐autonomic patients) (Rankin, 1957); and MAS score of 3 or 4/4 in any joint of the four limbs (representing severe spasticity) (Bohannon & Smith, 1987). A total of 31 patients fulfilled the inclusion criteria on admission to Hochzirl Hospital, where all patients were treated as in‐patients throughout the observation period. Four more patients, who were treated before implementation of the register, were recalled and added by one of the authors. To document improvement in awareness and cognitive abilities, those patients were identified who presented with a change of 2 or more points on the CRS‐R 6 months after implantation. We chose a 2‐point cutoff based on a previous study by Margetis et al. (2014), who attempted to differentiate spontaneous fluctuations in their patients' arousal levels from “true” changes. This led to exclusion of nine patients from statistical analysis: five of them did not improve by 2 or more points on the CRS‐R, three had already achieved a high functional level, and had already emerged from MCS at the time of admission (CRS‐R ≥ 20), rendering further improvement by 2 points impossible; one patient died unrelated to ITB shortly after pump implantation (Figure 1).

The following data were collected: patient age, gender, clinical diagnosis, and etiology at the time of admission (PRE); time intervals between acute event, PRE and pump implantation; daily ITB dosage, MAS, CRS‐R total and subscale scores at PRE and 3 and 6 months after pump implantation (3M, 6M), and changes over time (PRE to 3M and 3M to 6M, respectively) for MAS, CRS‐R total and CRS subscale scores, respectively. Collection of more data at additional time intervals would have been desirable, but due to their retrospective acquisition, the data would have been even more inhomogeneous and often missing. Therefore, only these three time points were finally chosen. Due to the retrospective nature of data, no ethics committee approval was obtained. The work was done in accordance with the Declaration of Helsinki.

Statistical analysis of patient characteristics aimed at identifying contributing factors. Specifically, we were interested whether an ITB‐associated reduction in spasticity would be associated with an improvement in consciousness, and if so in which domains in particular.

Statistical analysis was performed with Statistical Package for Social Sciences for Windows (SPSS Inc., Version 21.0). Non‐parametric Friedman test was applied to test for main effects of time on MAS, CRS‐R total, and subscale scores. Significant effects were followed up by post‐hoc Wilcoxon signed rank test. Spearman's rank‐order correlation test was applied to assess possible relations between CRS‐R total and subscale scores to ITB dosage and MAS scores at 3M and 6M, as well as on respective changes over time of CRS‐R total and subscale scores and MAS scores from PRE to 3M and from 3M to 6M, respectively. CRS‐R scores at 3M and 6M were also correlated to the time interval between acute event and pump implantation in order to assess whether “disease duration without ITB” might have an impact on the CRS‐R scores later on. We assumed that if time played a major role in gaining spontaneous recovery, then more time would be associated with more recovery, whereas if “duration of disease” played no role, then there should be no such correlation to CRS‐R scores.

Finally, further exploratory statistics were performed to identify potential influences of etiology, clinical state, and severity of DOC based on CRS‐R scores on admission. Patients were categorized according to etiology (group 1: TBI; group 2: non‐TBI, i.e., hypoxia and strokes), clinical diagnosis (group 1: UWS; group 2: MCS) (Giacino et al., 2002; The Multi‐Society Task Force on PVS, 1994a), and severity based on CRS‐R scores (group 1: CRS‐R < 7; group 2: CRS‐R > 7). Often different rehabilitation courses and outcomes are observed for patients with TBI vs. non‐TBI; the comparison of UWS vs. MCS patients was motivated by often encountered end‐of‐treatment discussions despite occasional observations of improvement in even very severely affected patients; the distinction in patients with CRS‐R scores below and above 7 was applied to confirm or refute that less or more affected patients would differ in their progress in the context of ITB treatment.

Friedman, Wilcoxon, and Spearman's rank‐order correlation tests were repeated in these subgroups. Mann–Whitney *U*‐test was used for non‐parametric data to test for differences between respective patient subgroups.

Due to the explorative nature of the study, uncorrected *p*‐values < .05 were considered statistically significant in a two‐tailed model for the total group analysis. Bonferroni correction was applied for multiple testing in subgroup analyses (*p* < .017 considered statistically significant).

## 3 RESULTS

### 3.1 All patients—CRS‐R total and subscale scores

Table 1 depicts patient demographics and characteristics. Friedman test revealed a significant effect of time of evaluation on CRS‐R total scores, each of the six CRS‐R subscale scores, and MAS scores. Largest CRS‐R score improvements occurred up to 3 months after pump implantation. MAS scores declined significantly from PRE to 3M, but not thereafter (Table 1, Table S1).

**TABLE 1:** Patient demographics and clinical characteristics of 26 DOC patients with consciousness recovery following ITB pump implantation

Sex: Male/female	(*n* [%])	16 (62%)/10 (38 %)
Age	[years, mean ± SD]	28.1 ± 14.2
Etiology		
Traumatic brain injury	(*n* [%])	19 (73%)
Cerebral hypoxia	(*n* [%])	2 (8%)
Subarachnoidal hemorrhage	(*n* [%])	3 (12%)
Intracerebral hemorrhage	(*n* [%])	2 (8%)
Time from event to admission	(months, median [range])	3.8 (0.9–40.8)
Time from admission to pump implantation	(days, median [range])	71 (17–694)
Clinical diagnosis pre pump implantation		
Unresponsive wakefulness syndrome	(*n* [%])	19 (73%)
Minimally conscious state	(*n* [%])	7 (27%)
Emergence from MCS	(*n* [%])	0 (0%)
Clinical diagnosis post pump implantation		
Unresponsive wakefulness syndrome	(*n* [%])	3 (12%)
Minimally conscious state	(*n* [%])	17 (65%)
Emergence from MCS	(*n* [%])	6 (23%)
CRS‐R prepump implantation	[mean ± SD]	6.9 ± 2.5^a^
CRS‐R 3 months post pump implantation	[mean ± SD]	11.8 ± 3.0^a^
CRS‐R 6 months post pump implantation	[mean ± SD]	13.3 ± 4.2^a^
MAS pre pump implantation	[mean ± SD]	3.9 ± 0.3^b^
MAS 3 months post pump implantation	[mean ± SD]	2.2 ± 0.5^b^
MAS 6 months post pump implantation	[mean ± SD]	2.1 ± 0.4^b^
ITB at 3 months post pump implantation	(μg, median [range])	245 (130–1050)
ITB at 6 months post pump implantation	(μg, median [range])	245 (150–1100)
oral baclofen pre pump implantation	(mg, median [range])	75 (37.5–100)
oral baclofen 3‐ and 6 months post pump implantation	(mg, median [range])	–
oral diazepam pre pump implantation	Patient #15 [mg]	15
oral diazepam 6 months post pump implantation	Patient #20 [mg]	10
s.c./i.v. clonidine pre pump implantation	Patients #15, 16, 18, 19, 24	0.3–0.45 mg/d s.c. 0.08 mg/h i.v.
s.c./i.v. clonidine 3‐ and 6 months post pump implantation		–

*Abbreviations*: CRS‐R, Coma Recovery Scale‐revised; DOC, disorder of consciousness; ITB, intrathecal baclofen; MCS, minimally conscious state; MAS, Modified Ashworth Score; SD, standard deviation.

^a^ Friedman: *p* = .000; post‐hoc Wilcoxon: *p* = .000 (PRE to 3M), *p* = .000 (3M to 6M).

^b^ Friedman: *p* = .000; post‐hoc Wilcoxon: *p* = .000 (PRE to 3M), *p* = .157 (3M to 6M).

Table 2 shows the results of correlation analysis of CRS‐R total scores, CRS‐R subscale scores, MAS scores, and daily ITB dosage, and of their respective changes over time (PRE to 3M and 3M to 6M). Significant correlations were only found for the changes over time of CRS‐R total and subscale scores between the two time periods, indicating that improvement early on (PRE to 3M) was significantly associated with later improvement (3M to 6M) during the observation period. Notably, however, neither CRS‐R total scores, nor subscale scores, nor their changes over time, correlated to corresponding MAS scores or their decline over time, indicating that a mere reduction in spasticity was not significantly associated with improvement in CRS‐R scores. Neither CRS‐R total scores nor subscale scores correlated to corresponding daily ITB doses, which varied widely in the studied patients (Table 1). There was also no correlation of CRS‐R total scores, nor CRS‐R subscale scores, nor their respective changes from one point in time to the next, with the time interval between acute event and pump implantation, concurring with no significant influence of time without ITB (i.e., reflecting spontaneous recovery) on later CRS‐R score developments (Table 2).

**TABLE 2:** Results of correlation analysis of Coma Recovery Scale‐revised (CRS‐R) total scores, CRS‐R subscale scores, Modified Ashworth Scale (MAS) scores, and daily dosage of intrathecal baclofen (ITB) in all 26 DOC patients with consciousness recovery following ITB pump implantation. PRE, on admission; 3M, 3 months after pump implantation; 6M, 6 months after pump implantation. Bold numbers indicate statistically significant correlations

Correlation	*ρ*	*p*
CRS‐R total score changes (PRE to 3M) to (3M to 6M)	**.433**	**.027**
CRS‐R total score to MAS score (3M)	.126	.540
CRS‐R total score to MAS score (6M)	−.107	.605
CRS‐R total score change to MAS score change (PRE to 3M)	−.065	.751
CRS‐R total score change to MAS score change (3M to 6M)	.010	.961
CRS‐R total score to daily ITB dose (3M)	.151	.462
CRS‐R total score to daily ITB dose (6M)	.160	.434
CRS‐R total score change (PRE to 3M) to daily ITB dose (3M)	−.068	.743
CRS‐R total score change (3M to 6M) to daily ITB dose (6M)	.091	.658
CRS‐R total score (3M) to time interval (acute event–pump implantation)	.129	.529
CRS‐R total score (6M) to time interval (acute event–pump implantation)	.106	.607
CRS‐R total score change (PRE to 3M) to time interval (acute event–pump implantation)	−.107	.602
CRS‐R total score change (3M to 6M) to time interval (acute event–pump implantation)	.019	.928
CRS‐R subscale score changes (PRE to 3M) to (3M to 6M)	**.736** to **.923**	**all < .000**
CRS‐R subscale scores to MAS score (3M)	.000 to .242	all > .234
CRS‐R subscale scores to MAS score (6M)	−.177 to −.074	all > .388
CRS‐R subscale score changes to MAS score change (PRE to 3M)	−.282 to .148	all > .163
CRS‐R subscale score changes to MAS score change (3M to 6M)	−.137 to .192	all > .346
CRS‐R subscale scores to daily ITB dose (3M)	−.256 to .322	all > .109
CRS‐R subscale scores to daily ITB dose (6M)	−.035 to .327	all > .103
CRS‐R subscale score changes (PRE to 3M) to daily ITB dose (3M)	−.267 to .087	all > .187
CRS‐R subscale score changes (3M to 6M) to daily ITB dose (6M)	−.094 to .173	all > .399
CRS‐R subscale scores (3M) to time (acute event–pump implantation)	−.184 to .275	.174 to .916
CRS‐R subscale scores (6M) to time (acute event–pump implantation)	−.225 to .322	.109 to .652
CRS‐R subscale scores changes (PRE to 3M) to time (acute event–pump implantation)	−.169 to .107	.410 to .864
CRS‐R subscale scores changes (3M to 6M) to time (acute event–pump implantation)	−.189 to .253	.212 to .920
MAS score to daily ITB dose (3M)	.194	.343
MAS score to daily ITB dose (6M)	.070	.733
MAS score change (PRE to 3M) to daily ITB dose (3M)	−.043	.869
MAS score change (3M to 6M) to daily ITB dose (6M)	−.231	.255

**FIGURE 2:**
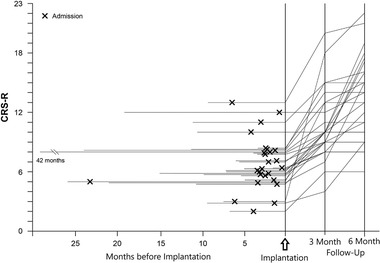
Evolution of CRS‐R total scores in 26 patients showing improvement following initiation of intrathecal baclofen treatment. “x” indicates time of admission and assessment of baseline CRS‐R scores. Further assessments were obtained at 3 and 6 months following pump implantation. Individual CRS‐R scores were extrapolated from the time of admission to pump implantation, because due to the retrospective nature of the study, CRS‐R scores were not obtained regularly for all patients at the time of implantation. No CRS‐R scores were obtained at the time of the acute event, of course, but the line was merely extrapolated from the one recorded at admission to visualize the intervals between acute event, admission, and pump implantation for each patient. Patients are synchronized according to pump implantation

**FIGURE 3:**
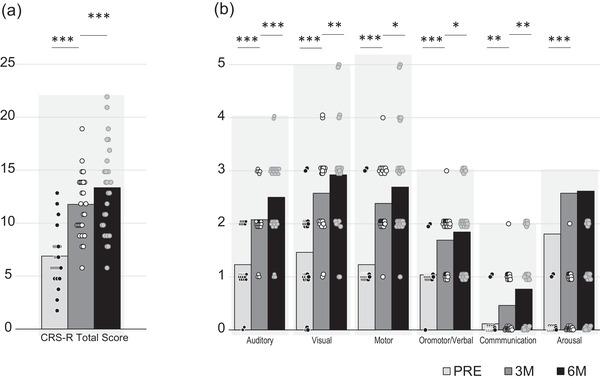
CRS‐R total scores (a) and CRS‐R subscale scores (b) in all patients at the time of admission (PRE) and 3 and 6 months after pump implantation (3M, 6M, respectively). The columns indicate mean values, and the dots represent individual patient data. The gray shades in the background of each set of three columns depicts the respective maximum number of the CRS‐R total score (i.e., 22), as well as the subscale scores (auditory: 4; visual: 5; motor: 6; oromotor/verbal: 3; communication: 2; arousal: 3). *p < .05; **p < .01; ***p < .001

Figure 2 shows the development of CRS‐R total scores of individual patients over time. Figures 3a and 3b depict mean and individual CRS‐R total scores and CRS‐R subscale scores, respectively, at time points PRE, 3M, and 6M.

### 3.2 Patient subgroups—CRS‐R total and subscale scores

To identify possible differences in the recovery of certain subgroups, patients were categorized according to etiology, clinical diagnosis, and severity based on initial CRS‐R scores. Table S2 depicts demographic data and characteristics of the respective patient subgroups. Friedman testing revealed a significant effect of time of evaluation on CRS‐R total scores and MAS scores in each patient subgroup, and on most but not all CRS‐R subscale scores (Table S3).

CRS‐R total scores improved in all subgroups from PRE to 3M, and in all but the non‐TBI and MCS subgroups from 3M to 6M. Like in the whole patient group, MAS scores declined significantly in all subgroups from PRE to 3M, but in no subgroup from 3M to 6M.

**FIGURE 4:**
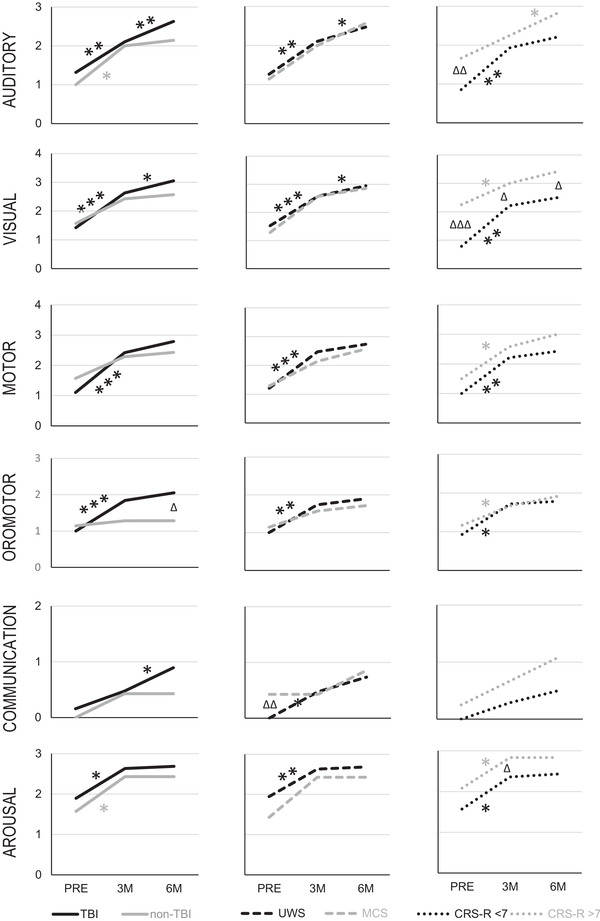
CRS‐R subscale scores in patient subgroups. * denotes significant difference within a patient subgroup between examination time points; ^Δ^ denotes significant difference between respective patient subgroups at a given time point. *p < .017; **p < .003; ***p < .0003; ^Δ^ *p* < .017; ^ΔΔ^ *p* < .003; ^ΔΔΔ^ *p* < .0003

The evolution over time of each CRS‐R subscale score in each patient subgroup, and differences between subgroups, are depicted in Figure 4. Respective statistical results of post‐hoc testing are shown in Table S3.

In the TBI subgroup, all but the communication subscale scores improved significantly from PRE to 3M, while only auditory, visual and communication subscale scores improved significantly from 3M to 6M. In the non‐TBI group, only auditory and arousal showed significant improvements from PRE to 3M, and no subscale score thereafter.

In the UWS subgroup, all subscale scores showed significant improvement from PRE to 3M, while only auditory and visual subscale scores improved significantly from 3M to 6M (with communication trending). In the MCS group, no significant changes were noted any time.

In the CRS‐R < 7 group, all but the communication subscale scores improved significantly from PRE to 3M, but none thereafter. In the CRS‐R > 7 group, all but auditory and communication subscale scores improved significantly from PRE to 3M, and the auditory subscale score from 3M to 6M (with visual and communication subscale scores trending).

Correlation analysis of relevant subgroup data is shown in Table S4. There were hardly any significant correlations. Similar to the total patient sample, CRS‐R total score changes correlated over time (from PRE–3M to 3M–6M) in the UWS and CRS‐R < 7 subgroups, possibly indicating that the patients who improved early on also improved later in these two subgroups. Notably, in no patient subgroup, there was a significant correlation of CRS‐R total score changes with MAS scores or respective MAS score changes at any time (Table S4). Interestingly, daily ITB dose at the 3‐month visit correlated significantly to corresponding changes (PRE to 3M) in CRS‐R total score and CRS‐R arousal subscale score in the non‐TBI subgroup, and in CRS‐R oromotor/verbal subscale score in the UWS subgroup.

Most differences between patient subgroups were noted in CRS‐R < 7 vs. CRS‐R > 7 subgroups, with CRS‐R total score and visual subscale score at all three time points, auditory subscale score at PRE, and arousal at 3M. TBI and non‐TBI groups differed significantly only in oromotor/verbal subscale at 6M, and UWS vs. MCS only in communication subscale at PRE (Table S2, Figure 4).

## 4 DISCUSSION

In the present study, in 26 patients with severe supraspinal spasticity treated with ITB, the largest improvements in both CRS‐R total and subscale scores as well as MAS scores were evident at the 3‐month visit after pump implantation. In contrast, after 6 months, further CRS‐R score improvements were smaller, while MAS scores showed no further decline. Notably, CRS‐R total and subscale scores did not correlate significantly to MAS scores at any point in time, and neither did score changes over time correlate to each other. These findings indicate that a mere reduction in spasticity was likely not the reason for patients to improve in awareness and cognitive abilities, thus rejecting our hypothesis. In the following sections, we discuss our findings in more detail and strive to elaborate on possible mechanisms underlying the observed effects on improved awareness and cognitive abilities, which we believe are related to baclofen, but not necessarily to its antispastic action.

### 4.1 Total patient group

Within 3 months after pump implantation, daily ITB dose was optimized to achieve good antispastic control as evidenced by a significant drop in MAS scores, which did not decline further at the 6‐month visit. This observation concurs well with previously published data in patients with supraspinal spasticity (Margetis et al., 2014; Natale et al., 2012; Posteraro et al., 2013; Rifici et al., 1994). MAS scores did not correlate to ITB dosage in the current patient group, perhaps due to the known large interindividual variability in response to ITB (Becker et al., 1997; Hoarau et al., 2012a; Maneyapanda et al., 2017; Margetis et al., 2014; Posteraro et al., 2013). Daily ITB dosage did not correlate to CRS‐R total scores in the total patient group, implicating no simple relationship between ITB and consciousness.

Three months after pump implantation, that is, after achieving good antispastic control, also CRS‐R scores improved significantly, underscoring a predominantly pharmacologically driven effect on both spasticity and cognition. However, the lack of correlation of CRS‐R scores to MAS scores, as well as of their respective changes over time, concurs with a previous report refuting a direct relationship between alleviation of spasticity and recovery of consciousness (Sarà et al., 2009). In selected patients, however, an ITB‐related reduction in spasticity per se might have possibly contributed to some improvement in awareness and cognitive functions. To this end, in children with cerebral palsy, selective dorsal rhizotomy and subsequent relief of leg spasticity have been reported to induce beneficial effects on higher brain functions (Craft et al., 1995).

The time interval between acute event and pump implantation differed widely among patients, ranging from 1.5 to 42 months, concurring with previous reports (Becker et al., 1997; Francisco et al., 2007; Margetis et al., 2014; Sarà et al., 2009). Spontaneous recovery may have contributed to or caused the patients’ improvement. However, the time interval between acute event and pump implantation, that is, the period without ITB, did neither correlate to CRS‐R scores nor to score changes at 3 and 6 months, which we would have expected, if a longer time would have enabled more spontaneous recovery. Selected patients who were in a stable clinical condition, for example, three patients in UWS for 7, 10, and 24 months, respectively, who began to speak comprehensibly within 3 months after ITB initiation, also favor a drug‐associated effect rather than spontaneous remission. A similar observation of late ITB‐associated recovery, from UWS to being able to verbally communicate after almost a year following the acute event, was reported earlier in two patients (Sarà et al., 2009). In a previous study, global outcome did not differ significantly in patients receiving ITB treatment within 3 months vs. those being implanted after 3 months post‐injury (Posteraro et al., 2013). Furthermore, late recovery of consciousness during ITB treatment, as previously reported (Sarà et al., 2009), underscores that there is neither a clear dependence of the time since injury for ITB to foster recovery of consciousness, nor that a longer duration of UWS or MCS is always associated with a lower chance of recovery, as commonly believed.

In the whole patient group, all CRS‐R subscale scores improved significantly within the first 3 months following pump implantation. Later, smaller, but significant improvements were seen for all subscale scores except arousal, which was on average already close to the maximum at 3 months and did not increase further (Figure 3b). Obviously, arousal is an important early recovery feature, concurring with the previously reported selective increase in arousal early after pump implantation, followed by further improvement across CRS‐R subscales related to commands circumventing limb movements (i.e., visual pursuit) (Sarà et al., 2009).

Similar to the CRS‐R total score, none of the subscale scores, nor their changes from one time point to the next, correlated to MAS scores (or their changes), corroborating the finding that reduction in spasticity was not the primary cause for improved cognitive functions.

### 4.2 Patient subgroups

Additional exploratory analysis in patient subgroups unveiled several interesting findings. Most results with respect to changes in CRS‐R total and subscale scores, in MAS scores, and the lack of correlation of CRS‐R to MAS scores, and their changes, paralleled those in the total patient sample.

Unexpectedly, patients with TBI did not differ significantly from non‐TBI patients with respect to CRS‐R total or subscale scores (except oromotor/verbal subscale score at 6M) nor MAS scores at any time point, which is in contrast to current literature reporting more common recovery in TBI than in cerebral hypoxia (Hoarau et al., 2012b; The Multi‐Society Task Force on PVS, 1994b).

Patients with UWS showed significant improvements in all CRS‐R subscale scores, whereas MCS patients did not. This may in part be due to the small patient groups on one hand, but also due to the difficulty in correctly diagnosing UWS patients (Schnakers et al., 2009). As pointed out by Formisano et al. (2019) in their recent case report, some patients with UWS should on retrospect perhaps be better classified as “functional locked‐in syndrome.” This term was proposed to describe patients with a dissociation between extreme behavioral motor dysfunction and preserved higher cortical functions as identified by functional imaging techniques (Bruno et al., 2011). The only significant difference between the two groups was observed in the CRS‐R communication subscale at PRE (Figure 4).

Patients with initial CRS‐R scores < 7 and > 7 gained similar absolute score points over time, but those with scores > 7 required ultimately higher daily ITB doses than those with scores < 7 (Table S2). At any rate, the present data suggest that even severely affected patients with initial CRS‐R < 7, should not be apodictically precluded from this therapeutic option, as some may still show substantial improvement in the course of ITB treatment. The present findings also question arbitrarily defined “time limits of irreversibility” and corroborate the change in terminology from “persistent vegetative state” to “UWS” (Laureys et al., 2010).

In contrast to the lack of correlation of CRS‐R scores to daily ITB dose in the whole patient group, there was a significant correlation of ITB dose at 3 months to several scores in a few subgroups (Table S4), suggesting some influence attributable to ITB.

All subgroups showed improvement after 3 months in the CRS‐R subscale scores arousal, auditory, and visual, in line with improvement in domains particularly evading limb movements, which might be hindered by spasticity. These data concur well with the previous notion (Sarà et al., 2009) that the observed recovery is not merely the result of “unmasked” motor control brought about by a general reduction in spasticity, and point to the need for other explanations for the effect of baclofen on awareness and cognitive abilities.

### 4.3 Previously suggested mechanisms for the effect of ITB on consciousness

Baclofen is a GABA_B_ receptor agonist, and GABA receptors are abundant in the nervous system. The drug may therefore exert its effects in many neuronal systems simultaneously, and perhaps in opposite ways in some. Furthermore, in an injured brain, the resulting net effects may differ among patients and from those in a healthy brain. Generally, beneficial pharmacological effects on motor and cognitive abilities may be directly dose related and immediate (within hours) as a result of a drug's pharmacokinetic action. Such observations have occasionally been reported in these patients, for example, zolpidem (Whyte et al., 2014). Alternatively, pharmacological effects may appear delayed as a function of neuroplasticity phenomena, for example, in the case reported by Oyama et al. (2010), who observed no improvement with ITB bolus application but did so between 3 weeks and 3 months during continuous ITB infusion, or in three of eight patients reported by Margetis et al. (2014), who described first signs of improvement after more than 2 years.

Sarà et al. (2009) suggested two possible mechanisms serving to explain the seemingly paradoxical effect of the inhibitory GABA_B_ agonist baclofen on consciousness: first, a selective spinal segmental effect with subsequent centripetal neuronal modulation of the cortex; and second, a modulation of dysregulated sleep–wake cycles that may interfere with alertness and awareness (Sarà et al., 2009).

The authors based the first proposed mechanism on the assumption that an injured brain, unable to adequately cope with incoming signals, may be overflooded by afferent proprioceptive input caused by spasticity, thus interfering with the maintenance of consciousness. ITB may modulate impulse transmission from the spinal cord to the cortex at the spinal level via presynaptic inhibition and spinal inhibitory interneurons (Sarà et al., 2009). This mechanism would imply that only patients with spasticity would benefit from ITB.

The second hypothesis put forward by Sarà et al. (2009) is centered on the assumption that baclofen may reach the cortex following spinal application via cerebrospinal fluid flow and may modulate cerebral neurotransmission, which in turn favors functional restoration among cortico–thalamo–cortical connections, some of which involve GABA_A_ and GABA_B_‐ergic thalamic neurons (Laureys et al., 2000). In that case, both patients with and without spasticity could theoretically benefit from baclofen (Pistoia et al., 2010). There is ample evidence of ITB effects at the cortical level following spinal application: reduced vigilance and status epilepticus following inadvertent drug overdose (Kofler et al., 1992), seizures following changes in ITB dosage (Kofler et al., 1994), and prolongation of the cortical silent period following ITB bolus application (Stetkarova & Kofler, 2013).

### 4.4 Other possible explanations for the effect of ITB on consciousness

ITB treatment may allow for reduction or even cessation of a wide range of systemic antispastic medication, thereby exerting a beneficial effect by limiting or alleviating their often‐sedating adverse effects. All patients were treated with oral and/or systemic antispastics, which were discontinued in all but one patient after ITB‐pump implantation (Table 1). However, personal experience suggests that occasional patients may show cognitive improvement under ITB even before oral medication is slowly tapered after pump implantation.

Recent findings have opened additional avenues possibly serving to explain how ITB may influence consciousness. There is no doubt that baclofen acts at the spinal and at the cortical level (Stetkarova & Kofler, 2013; Stokic et al., 2006). However, ample evidence has accumulated of an ITB action also at the brainstem level. Investigation of brainstem reflexes provided evidence of ITB‐related modulation of auditory startle reaction (Kumru et al., 2009), trigeminal blink reflex, and its modulation by paired pulses and by prepulses (Kumru et al., 2009, 2010, 2011), and masseteric inhibitory reflex (Kumru et al., 2009) in patients with spinal cord injury. The time course of effects following spinal bolus application with parallel modulation of blink reflex parameters and of muscle tone as assessed by MAS pointed to a direct pharmacokinetic brainstem action of ITB on tonic spasticity (Kumru et al., 2011). We therefore propose the brainstem as “missing link” between suggested ITB‐related actions at the spinal and at the cortical level.

The pedunculopontine nucleus (PPN) plays an important role in this context, as it is involved in prepulse inhibition (PPI), postural control, and arousal (Garcia‐Rill et al., 2019). PPI is considered an operational measure of sensorimotor gating (Swerdlow et al., 2016), which serves to screen or “to gate” external (sensory) and internal (cognitive, motor) information from higher order processing and subsequent responses, presumably to enable uninterrupted processing of the most salient aspects of the external and internal environment. More profound PPI reflects a narrower filter, that is, a more profound “gating out” of irrelevant information, and thus “freeing the brain” for the processing of more relevant stimuli. ITB was found to increase PPI (Kumru et al., 2011, 2010); thus, it is conceivable that ITB may contribute to the suppression of afferent information flow to the brain via PPI at the brainstem level.

The PPN is also involved in the control of posture, which is intertwined with the control of arousal. In neurorehabilitation, one of the main goals is verticalization of patients to render them most awake and alert. Notably, posture and origin of sensory input exert modulatory influence on PPI (Versace et al., 2019). Thus, ITB may theoretically act twofold: it may reduce spasticity in selected patients to a degree that allows for their verticalization, which had possibly been prevented by severe spasticity, prolonged bedrest, and subsequent “cardiocirculatory instability”; verticalization per se presumably contributes to arousal and thus better awareness, which may foster further rehabilitation progress; in addition, ITB may increase PPI, which was found for stimuli from the lower extremities more pronounced in the upright compared to supine posture (Versace et al., 2019).

The PPN contains cholinergic, glutamatergic, and GABAergic neurons. In experimental studies, GABAergic PPN neurons reached maximal activity during REM sleep, although some were also active during waking. PPN is involved in sleep–wake control through electrical coupling between GABAergic cortical interneurons, thalamic reticular neurons, and inferior olive neurons (Garcia‐Rill et al., 2019). The complex interrelationship of PPN, PPI, and sleep–wake control is also supported by findings of reduced PPI of the blink reflex in narcolepsy–cataplexy, in line with deficient orexin projections from the hypothalamus to the PPN (Frauscher et al., 2012).

The contribution of neurophysiological studies of neural brainstem circuits in patients who improve their level of consciousness after application of ITB would be decisive in corroborating this hypothesis. Serial investigation of PPI would be of greatest interest. Furthermore, the hand‐blink reflex, a defensive reflex mediated by brainstem circuits, which is modulated by cognitive influences (Versace et al., 2021), would be a suitable tool to track changes in consciousness induced at the brainstem level.

Another possible and less discussed route of action of ITB contributing to improved awareness and cognition may be an ITB‐related reduction in pain transmission. At the spinal level, there is evidence of an inhibitory action of ITB on nociceptor activity via presynaptic action at GABA_B_ receptors located on small‐diameter nociceptive afferents reaching the superficial dorsal horn (Riley et al., 2000). ITB exerted clinical analgesic effects on neuropathic pain in spinal cord injury patients both in open as well as randomized placebo‐controlled trials (Kumru et al., 2018, 2013), with long‐lasting benefit (Kumru et al., 2020). Similar to proprioceptive information overflow, nociceptive information overflow may disturb brain function, as self‐awareness may be influenced by concurrent stimuli which are normally modulated by gating mechanisms at the spinal and/or thalamic level (Melzack, 1999). Hence, suppressing nociceptive signaling at the spinal level may contribute to “liberating the brain” from irrelevant information overload, and to “freeing resources” for the processing of relevant stimuli.

In that line, also TBI‐associated dysautonomia, characterized by profuse sweating, tachycardia, hypertension, hyperthermia, tachypnea, hypersalivation, and bronchial hypersecretion, that is, “autonomic nervous system overflow,” was found to be negatively associated with recovery of awareness, while ITB‐associated alleviation of dysautonomia was associated with improved consciousness (Hoarau et al., 2012a).

Interestingly, experimental evidence suggests that electrical spinal cord stimulation, which is also applied for pain treatment, exerts its analgesic effect predominantly via the spinal GABA_B_ system (Cui et al., 1996). ITB enhanced the analgesic effect of spinal cord stimulation when applied simultaneously (Lind et al., 2008). In a prospective, uncontrolled, nonrandomized, observational study, spinal cord stimulation was reported to improve “awareness of self and surrounding” in 109 of 201 patients severely affected by TBI, cerebral hypoxia, and/or stroke (Kanno et al., 2009).

At the cerebral level, according to the “GABA impairment hypothesis” (Pistoia et al., 2014), GABA is necessary to switch from the resting state to goal‐oriented activities. “Switching off” the default mode network is a GABAergic process, which is impaired in patients with disorders of consciousness. Drugs like baclofen or zolpidem (Whyte et al., 2014) might in some patients partially reverse such a condition of impaired cortical GABA neurotransmission by rebalancing an increased ratio of synaptic excitation to synaptic inhibition, restoring the functional interplay between networks linking wakefulness and consciousness, and thereby exerting their seemingly paradoxical effects on consciousness (Pistoia et al., 2014).

### 4.5 Limitations

The biggest limitation is the retrospective single‐center study design, thus only few data points in time could be collated for valid comparison, while other data were merely extrapolated. However, as pointed out in several previous publications, unfortunately, prospective randomized placebo‐controlled multicenter trials are not feasible with ITB, as this treatment option is considered the only effective modality for severe generalized spasticity thus precluding placebo‐controlled studies.

Another limitation may be the large range of intervals from the acute event to the time of pump implantation. However, there was no correlation of these intervals to the CRS‐R scores obtained during ITB treatment, in accordance with previous research by others.

Although this report is the largest so far focusing on the early recovery of awareness and cognitive abilities in patients suffering from severe supraspinal spasticity following initiation of ITB treatment, the total number of patients is still small, and diagnoses are heterogeneous and not evenly distributed.

No functional MRI studies were applied aiming at differentiating UWS from functional locked‐in patients.

Finally, comorbidities were not assessed but may have considerably influenced the timing of pump implantation and/or the course of recovery in selected patients. In particular, urinary and pulmonary infections likely occurred across all ages and etiologies.

## 5 CONCLUSION

Our observations corroborate and extend previous findings on the recovery of awareness and cognitive abilities in association with ITB treatment in few previously reported patients. The observed effects 3 months after pump implantation render a pharmacologically driven mechanism more likely than mere “spontaneous recovery.” However, the beneficial effect of ITB on awareness and cognitive functions seems to be independent of its antispastic action. Underlying neural processes explaining the observed improvements remain unclear and speculative. Based on recent findings of ITB‐related effects at the brainstem level, we propose the brainstem as “missing link” between suggested ITB‐related actions at the spinal and at the cortical level. At any rate, given the heterogeneity of patients and of their cerebral lesions, particularly with TBI and stroke, it is likely that different mechanisms—possibly in parallel—may be responsible for the occasionally observed improvement in awareness and cognitive abilities in patients treated with ITB.

Future research should strive to elaborate on possible mechanisms of recovery of consciousness and cognition in patients receiving ITB by combining neurophysiology, for example, brainstem reflexes modulation, high‐density electroencephalography, power spectral and brain network analysis, with functional brain imaging.

## CONFLICT OF INTEREST

No conflict of interest has been declared by the authors.

### PEER REVIEW

The peer review history for this article is available at https://publons.com/publon/10.1002/brb3.2566

## Supporting information



Supporting Information

Supporting Information

Supporting Information

Supporting Information

## Data Availability

Raw data were generated at LKH Hochzirl Department of Neurology. Derived data supporting the findings of this study are available from the corresponding author E.P. on request.
