# Supplementary material for: On the recovery of disorders of consciousness under intrathecal baclofen administration for severe spasticity—An observational study
Source: Brain Behav. 2022 Apr 10;12(5):e2566. doi: 10.1002/brb3.2566 (PMC9120732; doi:10.1002/brb3.2566)
Supplement: Supplementary file 1 — Supporting Information [file BRB3-12-e2566-s003.docx]

Supplementary Table 1. Results of statistical analysis of Coma Recovery Scale-revised (CRS-R) total and subscale scores in all patients. PRE, on admission; 3M, 3 months after pump implantation; 6M, 6 months after pump implantation.

|  | Friedman | Post-hoc Wilcoxon  PRE – 3M | Post-hoc Wilcoxon  3M – 6M |
| --- | --- | --- | --- |
|  |  |  |  |
|  |  |  |  |
| CRS-R total score | **.000** | **.000** | **.000** |
|  |  |  |  |
| CRS-R auditory | **.000** | **.000** | **.001** |
| CRS-R visual | **.000** | **.000** | **.003** |
| CRS-R motor | **.000** | **.000** | **.011** |
| CRS-R oromotor/verbal | **.000** | **.000** | **.046** |
| CRS-R communication | **.000** | **.007** | **.005** |
| CRS-R arousal | **.000** | .**000** | .317 |
|  |  |  |  |
|  |  |  |  |
