# Supplementary material for: On the recovery of disorders of consciousness under intrathecal baclofen administration for severe spasticity—An observational study
Source: Brain Behav. 2022 Apr 10;12(5):e2566. doi: 10.1002/brb3.2566 (PMC9120732; doi:10.1002/brb3.2566)
Supplement: Supplementary file 2 — Supporting Information [file BRB3-12-e2566-s004.docx]

Supplementary Table 2. Patient subgroup characteristics according to etiology, clinical diagnosis, and initial Coma Recovery Scale-revised (CRS-R) score.

|  |  |  |  |  |  |  |  |
| --- | --- | --- | --- | --- | --- | --- | --- |
|  |  | Etiology | | Clinical diagnosis | | CRS-R score on admission | |
|  |  | TBI | non-TBI | UWS | MCS | < 7 | > 7 |
|  |  |  |  |  |  |  |  |
|  |  |  |  |  |  |  |  |
| Sex: Male / Female | [number (%)] | 14 (74%) / 5 (26%) | 5 (71%) / 2 (29%) | 13 (68%) / 6 (32%) | 3 (43%) / 4 (57%) | 7 (50%) / 7 (50%) | 9 (75%) / 3 (25%) |
| Age | [years, mean ± SD] | 24.7 ± 10.7 | 37.3 ± 18.8 | 25.7 ± 10.0 | 34.6 ± 21.2 | 31.5 ± 15.6 | 24.1 ± 11.7 |
|  |  |  |  |  |  |  |  |
| Time from event to admission | [months, median (Range)] | 4.0 (0.9 - 40.8) | 3.7 (2.7 - 18.3) | 4.0 (0.9 - 40.8) | 2.8 (1.3 - 9,6) | 3.3 (1.0 - 17.6) | 5.6 (0.9 - 40.8) |
| Time from admission to pump implantation | [days, median (Range)] | 77 (20 - 694) | 62 (17 - 192) | 70 (17 - 694) | 77 (20 - 192) | 73 (20 - 192) | 70 (17 - 694) |
|  |  |  |  |  |  |  |  |
| Clinical diagnosis pre pump implantation |  |  |  |  |  |  |  |
| UWS | [number (%)] | 14 (74%) | 5 (71%) | 19 (100%) | 0 (0%) | 10 (72%) | 9 (75%) |
| MCS | [number (%)] | 5 (26%) | 2 (29%) | 0 (0%) | 7 (100%) | 4 (28%) | 3 (25%) |
| Emergence from MCS | [number (%)] | 0 (0%) | 0 (0%) | 0 (0%) | 0 (0%) | 0 (0%) | 0 (0%) |
|  |  |  |  |  |  |  |  |
| Clinical diagnosis post pump implantation |  |  |  |  |  |  |  |
| UWS | [number (%)] | 2 (11%) | 1 (14%) | 3 (15%) | 0 (0%) | 2 (14%) | 1 (8%) |
| MCS | [number (%)] | 13 (68%) | 4 (57%) | 12 (63%) | 5 (71%) | 10 (72%) | 7 (58%) |
| Emergence from MCS | [number (%)] | 4 (21%) | 2 (29%) | 4 (21%) | 2 (29%) | 2 (14%) | 4 (33%) |
|  |  |  |  |  |  |  |  |
| CRS-R pre pump implantation * | [mean ± SD] | 6.9 ± 2.2 | 6.9 ± 3.5 | 6.9 ± 2.0 | 6.7 ± 3.9 | 5.1 ± 1.3 | 8.9 ± 2.1 |
| CRS-R 3-months post pump implantation * | [mean ± SD] | 12.1 ± 2.9 | 10.9 ± 3.2 | 12.0 ± 2.9 | 11.1 ± 3.4 | 10.7 ± 3.4 | 13.0 ± 1.9 |
| CRS-R 6-months post pump implantation * | [mean ± SD] | 14.1 ± 4.2 | 11.3 ± 3.7 | 13.5 ± 3.8 | 13.0 ± 5.5 | 11.9 ± 4.5 | 15.1 ± 3.3 |
|  |  |  |  |  |  |  |  |
| MAS pre pump implantation | [mean ± SD] | 3.9 ± 0.3 | 4.0 ± 0.0 | 3.9 ± 0.3 | 4.0 ± 0.0 | 4.1 ± 1.1 | 7.8 ± 2.1 |
| MAS 3-months post pump implantation | [mean ± SD] | 2.2 ± 0.4 | 2.1 ± 0.7 | 2.3 ± 0.5 | 2.0 ± 0.6 | 9.6 ± 4.4 | 10.9 ± 3.1 |
| MAS 6-months post pump implantation | [mean ± SD] | 2.2 ± 0.4 | 2.0 ± 0.6 | 2.2 ± 0.4 | 2.0 ± 0.6 | 13.1 ± 5.0 | 13.3 ± 3.6 |
|  |  |  |  |  |  |  |  |
| ITB at 3-months post pump implantation | [median (Range)] | 220 (130 - 1050) | 320 (150 - 410) | 250 (130 - 1050) | 200 (150 - 450) | 185 (130 - 450) | 325 (130 – 1050) |
| ITB at 6-months post pump implantation | [median (Range)] | 220 (150 - 1100) | 320 (150 - 608) | 260 (150 - 1100) | 220 (150 - 846) | 195 (150 - 846) | 305 (160 - 1100) |
|  |  |  |  |  |  |  |  |

CRS-R, Coma Recovery Scale-revised; TBI, traumatic brain injury; UWS, unresponsive wakefulness syndrome; MCS, minimally conscious state; SD, standard deviation; ITB, intrathecal baclofen

* Between-group comparison: CRS-R scores not significantly different between TBI and non-TBI groups, and between UWS and MCS groups at any time point; between CRS-R <7 and CRS-R >7 groups differences at PRE (P = .000), 3M (P = .021), and 6M (P = .037).

** Between-group comparison: MAS scores not significantly different between TBI and non-TBI groups, between UWS and MCS groups, and between CRS-R <7 and CRS-R >7 groups at any time point.
