# Supplementary material for: On the recovery of disorders of consciousness under intrathecal baclofen administration for severe spasticity—An observational study
Source: Brain Behav. 2022 Apr 10;12(5):e2566. doi: 10.1002/brb3.2566 (PMC9120732; doi:10.1002/brb3.2566)
Supplement: Supplementary file 3 — Supporting Information [file BRB3-12-e2566-s002.docx]

Supplementary Table 3. Results of statistical analysis in patient subgroups, indicating reduction in Modified Ashworth Scale (MAS) score, and improvement in Coma Recovery Scale-revised (CRS-R) total scores and CRS-R subscale scores. P-values <.017 significant after Bonferroni correction.

| Subgroup |  | Friedman | Post-hoc Wilcoxon  PRE – 3M | Post-hoc Wilcoxon  3M – 6M |
| --- | --- | --- | --- | --- |
|  |  |  |  |  |
|  |  |  |  |  |
| TBI | CRS-R total | **.000** | **.000** | **.001** |
| non-TBI |  | **.001** | **.017** | .180 |
|  |  |  |  |  |
| UWS |  | **.000** | **.000** | **.002** |
| MCS |  | **.002** | **.017** | .066 |
|  |  |  |  |  |
| CRS-R <7 |  | **.000** | **.001** | **.017** |
| CRS-R >7 |  | **.000** | **.002** | **.007** |
|  |  |  |  |  |
|  |  |  |  |  |
| TBI | MAS | **.000** | **.000** | .317 |
| non-TBI |  | **.001** | **.016** | .317 |
|  |  |  |  |  |
| UWS |  | **.000** | **.000** | .157 |
| MCS |  | **.001** | **.014** | 1.00 |
|  |  |  |  |  |
| CRS-R <7 |  | **.000** | **.001** | .317 |
| CRS-R >7 |  | **.000** | **.001** | .317 |
|  |  |  |  |  |
|  |  |  |  |  |
| TBI | CRS-R auditory | **.000** | **.002** | **.002** |
|  | CRS-R visual | **.000** | **.000** | **.005** |
|  | CRS-R motor | **.000** | **.000** | .020 |
|  | CRS-R oromotor/verbal | **.000** | **.000** | .046 |
|  | CRS-R communication | **.000** | .034 | **.005** |
|  | CRS-R arousal | **.000** | .**001** | .317 |
|  |  |  |  |  |
| non-TBI | CRS-R auditory | **.001** | **.008** | .317 |
|  | CRS-R visual | **.006** | .034 | .317 |
|  | CRS-R motor | **.015** | .059 | .317 |
|  | CRS-R oromotor/verbal | .368 | .317 | 1.00 |
|  | CRS-R communication | .050 | .083 | 1.00 |
|  | CRS-R arousal | **.001** | **.016** | .317 |
|  |  |  |  |  |
|  |  |  |  |  |
| UWS | CRS-R auditory | **.000** | **.001** | **.008** |
|  | CRS-R visual | **.000** | **.000** | **.008** |
|  | CRS-R motor | **.000** | **.000** | .025 |
|  | CRS-R oromotor/verbal | **.000** | **.001** | .083 |
|  | CRS-R communication | **.000** | **.007** | .025 |
|  | CRS-R arousal | **.000** | **.002** | .317 |
|  |  |  |  |  |
| MCS | CRS-R auditory | **.003** | .034 | .046 |
|  | CRS-R visual | **.004** | .024 | .157 |
|  | CRS-R motor | **.006** | .034 | .180 |
|  | CRS-R oromotor/verbal | .039 | .083 | .317 |
|  | CRS-R communication | .050 | 1.00 | .083 |
|  | CRS-R arousal | .**007** | .038 | 1.000 |
|  |  |  |  |  |
|  |  |  |  |  |
| CRS-R <7 | CRS-R auditory | **.000** | **.001** | .046 |
|  | CRS-R visual | **.000** | **.001** | .046 |
|  | CRS-R motor | **.000** | **.001** | .083 |
|  | CRS-R oromotor/verbal | **.000** | **.005** | .317 |
|  | CRS-R communication | .018 | .102 | .083 |
|  | CRS-R arousal | **.000** | **.009** | .317 |
|  |  |  |  |  |
| CRS-R >7 | CRS-R auditory | **.000** | .020 | **.008** |
|  | CRS-R visual | **.000** | **.007** | .025 |
|  | CRS-R motor | **.000** | **.006** | .059 |
|  | CRS-R oromotor/verbal | **.002** | **.014** | .083 |
|  | CRS-R communication | **.002** | .025 | .025 |
|  | CRS-R arousal | **.000** | **.007** | 1.00 |
|  |  |  |  |  |
|  |  |  |  |  |

CRS-R, Coma Recovery Scale-revised; TBI, traumatic brain injury; UWS, unresponsive wakefulness syndrome; MCS, minimally conscious state;
