# Supplementary material for: On the recovery of disorders of consciousness under intrathecal baclofen administration for severe spasticity—An observational study
Source: Brain Behav. 2022 Apr 10;12(5):e2566. doi: 10.1002/brb3.2566 (PMC9120732; doi:10.1002/brb3.2566)
Supplement: Supplementary file 4 — Supporting Information [file BRB3-12-e2566-s001.docx]

Supplementary Table 4. Results of correlation analysis of Coma Recovery Scale-revised (CRS-R) total scores, CRS-R subscale scores, Modified Ashworth Scale (MAS) scores, and daily dosage of intrathecal baclofen (ITB) in 26 DOC patients with consciousness recovery following ITB pump implantation categorized in patient subgroups according to etiology, clinical diagnosis, and severity based on initial CRS-R scores.

|  |  |  |
| --- | --- | --- |
| Correlations | ρ | P |
|  |  |  |
|  |  |  |
| CRS-R total score changes (*PRE–3M*) to (*3M–6M*) TBI | .381 | .107 |
| CRS-R total score changes (*PRE–3M*) to (*3M–6M*) non-TBI | .233 | .615 |
| CRS-R total score changes (*PRE–3M*) to (*3M–6M*) UWS | **.580** | **.009** |
| CRS-R total score changes (*PRE–3M*) to (*3M–6M*) MCS | .000 | 1.00 |
| CRS-R total score changes (*PRE–3M*) to (*3M–6M*) CRS-R <7 | **.714** | **.004** |
| CRS-R total score changes (*PRE–3M*) to (*3M–6M*) CRS-R >7 | .351 | .263 |
|  |  |  |
|  |  |  |
| CRS-R total score changes (*PRE–3M*) to MAS score (*3M*) TBI | .251 | .299 |
| CRS-R total score changes (*3M–6M*) to MAS score (*6M*) TBI | -.349 | .143 |
| CRS-R total score changes (*PRE–3M*) to MAS score change (*PRE–3M*) TBI | .011 | .966 |
| CRS-R total score changes (*3M–6M*) to MAS score change (*3M–6M*) TBI | -.175 | .473 |
|  |  |  |
| CRS-R total score changes (*PRE–3M*) to MAS score (*3M*) non-TBI | -.658 | .108 |
| CRS-R total score changes (*3M–6M*) to MAS score (*6M*) non-TBI | -.000 | 1.00 |
| CRS-R total score changes (*PRE–3M*) to MAS score change (*PRE–3M*) non-TBI | .658 | .108 |
| CRS-R total score changes (*3M–6M*) to MAS score change (*3M–6M*) non-TBI | .255 | .582 |
|  |  |  |
|  |  |  |
| CRS-R total score changes (*PRE–3M*) to MAS score (*3M*) UWS | -.011 | .964 |
| CRS-R total score changes (*3M–6M*) to MAS score (*6M*) UWS | -.245 | .312 |
| CRS-R total score changes (*PRE–3M*) to MAS score change (*PRE–3M*) UWS | -.171 | .483 |
| CRS-R total score changes (*3M–6M*) to MAS score change (*3M–6M*) UWS | .000 | 1.00 |
|  |  |  |
| CRS-R total score changes (*PRE–3M*) to MAS score (*3M*) MCS | .350 | .441 |
| CRS-R total score changes (*3M–6M*) to MAS score (*6M*) MCS | .000 | 1.00 |
| CRS-R total score changes (*PRE–3M*) to MAS score change (*PRE–3M*) MCS | .350 | .441 |
| CRS-R total score changes (*3M–6M*) to MAS score change (*3M–6M*) MCS | -- | -- |
|  |  |  |
|  |  |  |
| CRS-R total score changes (*PRE–3M*) to MAS score (*3M*) CRS-R<7 | .336 | .239 |
| CRS-R total score changes (*3M–6M*) to MAS score (*6M*) CRS-R<7 | -.161 | .582 |
| CRS-R total score changes (*PRE–3M*) to MAS score change (*PRE–3M*) CRS-R<7 | .029 | .921 |
| CRS-R total score changes (*3M–6M*) to MAS score change (*3M–6M*) CRS-R<7 | -.370 | .193 |
|  |  |  |
| CRS-R total score changes (*PRE–3M*) to MAS score (*3M*) CRS-R>7 | -.230 | .471 |
| CRS-R total score changes (*3M–6M*) to MAS score (*6M*) CRS-R>7 | -.264 | .407 |
| CRS-R total score changes (*PRE–3M*) to MAS score change (*PRE–3M*) CRS-R>7 | -.230 | .471 |
| CRS-R total score changes (*3M–6M*) to MAS score change (*3M–6M*) CRS-R>7 | .400 | .197 |
|  |  |  |
|  |  |  |
| CRS-R total score (*3M*) to daily ITB dose (*3M*) TBI | .257 | .288 |
| CRS-R total score (*6M*) to daily ITB dose (*6M*) TBI | .303 | .207 |
| CRS-R total score changes (*PRE–3M*) to daily ITB dose (*3M*) TBI | .185 | .449 |
| CRS-R total score changes (*3M–6M*) to daily ITB dose (*6M*) TBI | .269 | .265 |
|  |  |  |
| CRS-R total score (*3M*) to daily ITB dose (*3M*) non-TBI | -.048 | .919 |
| CRS-R total score (*6M*) to daily ITB dose (*6M*) non-TBI | -.046 | .922 |
| CRS-R total score changes (*PRE–3M*) to daily ITB dose (*3M*) non-TBI | **-.819** | **.024** |
| CRS-R total score changes (*3M–6M*) to daily ITB dose (*6M*) non-TBI | -.249 | .590 |
|  |  |  |
|  |  |  |
| CRS-R total score (*3M*) to daily ITB dose (*3M*) UWS | .156 | .524 |
| CRS-R total score (*6M*) to daily ITB dose (*6M*) UWS | .137 | .576 |
| CRS-R total score changes (*PRE–3M*) to daily ITB dose (*3M*) UWS | -.206 | .397 |
| CRS-R total score changes (*3M–6M*) to daily ITB dose (*6M*) UWS | -.001 | .996 |
|  |  |  |
| CRS-R total score (*3M*) to daily ITB dose (*3M*) MCS | .091 | .846 |
| CRS-R total score (*6M*) to daily ITB dose (*6M*) MCS | .718 | .069 |
| CRS-R total score changes (*PRE–3M*) to daily ITB dose (*3M*) MCS | .468 | .290 |
| CRS-R total score changes (*3M–6M*) to daily ITB dose (*6M*) MCS | .663 | .105 |
|  |  |  |
|  |  |  |
| CRS-R total score (*3M*) to daily ITB dose (*3M*) CRS-R<7 | .055 | .853 |
| CRS-R total score (*6M*) to daily ITB dose (*6M*) CRS-R<7 | .043 | .883 |
| CRS-R total score changes (*PRE–3M*) to daily ITB dose (*3M*) CRS-R<7 | .058 | .843 |
| CRS-R total score changes (*3M–6M*) to daily ITB dose (*6M*) CRS-R<7 | .121 | .680 |
|  |  |  |
| CRS-R total score (*3M*) to daily ITB dose (*3M*) CRS-R>7 | -.007 | .982 |
| CRS-R total score (*6M*) to daily ITB dose (*6M*) CRS-R>7 | -.067 | .837 |
| CRS-R total score changes (*PRE–3M*) to daily ITB dose (*3M*) CRS-R>7 | .065 | .841 |
| CRS-R total score changes (*3M–6M*) to daily ITB dose (*6M*) CRS-R>7 | -.135 | .675 |
|  |  |  |
|  |  |  |
| CRS-R auditory change (*PRE–3M*) to daily ITB dose (*3M*) TBI subgroup | -.097 | .693 |
| CRS-R auditory change (*3M–6M*) to daily ITB dose (*6M*) TBI subgroup | .183 | .453 |
|  |  |  |
| CRS-R auditory change (*PRE–3M*) to daily ITB dose (*3M*) non-TBI subgroup | --- | --- |
| CRS-R auditory change (*3M–6M*) to daily ITB dose (*6M*) non-TBI subgroup | -.520 | .232 |
|  |  |  |
| CRS-R auditory change (*PRE–3M*) to daily ITB dose (*3M*) UWS subgroup | -.104 | .673 |
| CRS-R auditory change (*3M–6M*) to daily ITB dose (*6M*) UWS subgroup | -.140 | .569 |
|  |  |  |
| CRS-R auditory change (*PRE–3M*) to daily ITB dose (*3M*) MCS subgroup | .020 | .966 |
| CRS-R auditory change (*3M–6M*) to daily ITB dose (*6M*) MCS subgroup | .289 | .530 |
|  |  |  |
| CRS-R auditory change (*PRE–3M*) to daily ITB dose (*3M*) CRS-R <7 subgroup | .151 | .606 |
| CRS-R auditory change (*3M–6M*) to daily ITB dose (*6M*) CRS-R <7 subgroup | .197 | .499 |
|  |  |  |
| CRS-R auditory change (*PRE–3M*) to daily ITB dose (*3M*) CRS-R >7 subgroup | .019 | .952 |
| CRS-R auditory change (*3M–6M*) to daily ITB dose (*6M*) CRS-R >7 subgroup | -.564 | .056 |
|  |  |  |
|  |  |  |
| CRS-R visual change (*PRE–3M*) to daily ITB dose (*3M*) TBI subgroup | .101 | .681 |
| CRS-R visual change (*3M–6M*) to daily ITB dose (*6M*) TBI subgroup | .273 | .258 |
|  |  |  |
| CRS-R visual change (*PRE–3M*) to daily ITB dose (*3M*) non-TBI subgroup | -.436 | .328 |
| CRS-R visual change (*3M–6M*) to daily ITB dose (*6M*) non-TBI subgroup | .104 | .825 |
|  |  |  |
| CRS-R visual change (*PRE–3M*) to daily ITB dose (*3M*) UWS subgroup | -.247 | .308 |
| CRS-R visual change (*3M–6M*) to daily ITB dose (*6M*) UWS subgroup | .150 | .541 |
|  |  |  |
| CRS-R visual change (*PRE–3M*) to daily ITB dose (*3M*) MCS subgroup | .386 | .393 |
| CRS-R visual change (*3M–6M*) to daily ITB dose (*6M*) MCS subgroup | .158 | .735 |
|  |  |  |
| CRS-R visual change (*PRE–3M*) to daily ITB dose (*3M*) CRS-R <7 subgroup | .148 | .613 |
| CRS-R visual change (*3M–6M*) to daily ITB dose (*6M*) CRS-R <7 subgroup | .177 | .544 |
|  |  |  |
| CRS-R visual change (*PRE–3M*) to daily ITB dose (*3M*) CRS-R >7 subgroup | -.068 | .834 |
| CRS-R visual change (*3M–6M*) to daily ITB dose (*6M*) CRS-R >7 subgroup | .123 | .704 |
|  |  |  |
|  |  |  |
| CRS-R motor change (*PRE–3M*) to daily ITB dose (*3M*) TBI subgroup | .258 | .287 |
| CRS-R motor change (*3M–6M*) to daily ITB dose (*6M*) TBI subgroup | .065 | .792 |
|  |  |  |
| CRS-R motor change (*PRE–3M*) to daily ITB dose (*3M*) non-TBI subgroup | -.314 | .492 |
| CRS-R motor change (*3M–6M*) to daily ITB dose (*6M*) non-TBI subgroup | .104 | .825 |
|  |  |  |
| CRS-R motor change (*PRE–3M*) to daily ITB dose (*3M*) UWS subgroup | -.086 | .726 |
| CRS-R motor change (*3M–6M*) to daily ITB dose (*6M*) UWS subgroup | -.033 | .894 |
|  |  |  |
| CRS-R motor change (*PRE–3M*) to daily ITB dose (*3M*) MCS subgroup | -.139 | .766 |
| CRS-R motor change (*3M–6M*) to daily ITB dose (*6M*) MCS subgroup | .178 | .702 |
|  |  |  |
| CRS-R motor change (*PRE–3M*) to daily ITB dose (*3M*) CRS-R <7 subgroup | -.323 | .259 |
| CRS-R motor change (*3M–6M*) to daily ITB dose (*6M*) CRS-R <7 subgroup | .022 | .941 |
|  |  |  |
| CRS-R motor change (*PRE–3M*) to daily ITB dose (*3M*) CRS-R >7 subgroup | .179 | .578 |
| CRS-R motor change (*3M–6M*) to daily ITB dose (*6M*) CRS-R >7 subgroup | .-.118 | .715 |
|  |  |  |
|  |  |  |
| CRS-R oromotor/verbal change (*PRE–3M*) to daily ITB dose (*3M*) TBI subgroup | -.134 | .586 |
| CRS-R oromotor/verbal change (*3M–6M*) to daily ITB dose (*6M*) TBI subgroup | .142 | .563 |
|  |  |  |
| CRS-R oromotor/verbal change (*PRE–3M*) to daily ITB dose (*3M*) non-TBI subgroup | -.520 | .232 |
| CRS-R oromotor/verbal change (*3M–6M*) to daily ITB dose (*6M*) non-TBI subgroup | --- | --- |
|  |  |  |
| CRS-R oromotor/verbal change (*PRE–3M*) to daily ITB dose (*3M*) UWS subgroup | **-.474** | **.040** |
| CRS-R oromotor/verbal change (*3M–6M*) to daily ITB dose (*6M*) UWS subgroup | .198 | .417 |
|  |  |  |
| CRS-R oromotor/verbal change (*PRE–3M*) to daily ITB dose (*3M*) MCS subgroup | .289 | .530 |
| CRS-R oromotor/verbal change (*3M–6M*) to daily ITB dose (*6M*) MCS subgroup | ,000 | 1.00 |
|  |  |  |
| CRS-R oromotor/verbal change (*PRE–3M*) to daily ITB dose (*3M*) CRS-R <7 subgroup | -.043 | .883 |
| CRS-R oromotor/verbal change (*3M–6M*) to daily ITB dose (*6M*) CRS-R <7 subgroup | -.242 | .404 |
|  |  |  |
| CRS-R oromotor/verbal change (*PRE–3M*) to daily ITB dose (*3M*) CRS-R >7 subgroup | -.290 | .361 |
| CRS-R oromotor/verbal change (*3M–6M*) to daily ITB dose (*6M*) CRS-R >7 subgroup | .251 | .431 |
|  |  |  |
|  |  |  |
| CRS-R communication change (*PRE–3M*) to daily ITB dose (*3M*) TBI subgroup | .010 | .967 |
| CRS-R communication change (*3M–6M*) to daily ITB dose (*6M*) TBI subgroup | .263 | .276 |
|  |  |  |
| CRS-R communication change (*PRE–3M*) to daily ITB dose (*3M*) non-TBI subgroup | -.147 | .753 |
| CRS-R communication change (*3M–6M*) to daily ITB dose (*6M*) non-TBI subgroup | --- | --- |
|  |  |  |
| CRS-R communication change (*PRE–3M*) to daily ITB dose (*3M*) UWS subgroup | -.025 | .918 |
| CRS-R communication change (*3M–6M*) to daily ITB dose (*6M*) UWS subgroup | .055 | .824 |
|  |  |  |
| CRS-R communication change (*PRE–3M*) to daily ITB dose (*3M*) MCS subgroup | --- | --- |
| CRS-R communication change (*3M–6M*) to daily ITB dose (*6M*) MCS subgroup | .000 | 1.00 |
|  |  |  |
| CRS-R communication change (*PRE–3M*) to daily ITB dose (*3M*) CRS-R <7 subgroup | -.226 | .438 |
| CRS-R communication change (*3M–6M*) to daily ITB dose (*6M*) CRS-R <7 subgroup | .152 | .604 |
|  |  |  |
| CRS-R communication change (*PRE–3M*) to daily ITB dose (*3M*) CRS-R >7 subgroup | .220 | .491 |
| CRS-R communication change (*3M–6M*) to daily ITB dose (*6M*) CRS-R >7 subgroup | -.025 | .940 |
|  |  |  |
|  |  |  |
| CRS-R arousal change (*PRE–3M*) to daily ITB dose (*3M*) TBI subgroup | .435 | .063 |
| CRS-R arousal change (*3M–6M*) to daily ITB dose (*6M*) TBI subgroup | -.086 | .725 |
|  |  |  |
| CRS-R arousal change (*PRE–3M*) to daily ITB dose (*3M*) non-TBI subgroup | **-.608** | **.028** |
| CRS-R arousal change (*3M–6M*) to daily ITB dose (*6M*) non-TBI subgroup | --- | --- |
|  |  |  |
| CRS-R arousal change (*PRE–3M*) to daily ITB dose (*3M*) UWS subgroup | .011 | .964 |
| CRS-R arousal change (*3M–6M*) to daily ITB dose (*6M*) UWS subgroup | -.129 | .598 |
|  |  |  |
| CRS-R arousal change (*PRE–3M*) to daily ITB dose (*3M*) MCS subgroup | .000 | 1.00 |
| CRS-R arousal change (*3M–6M*) to daily ITB dose (*6M*) MCS subgroup | --- | --- |
|  |  |  |
| CRS-R arousal change (*PRE–3M*) to daily ITB dose (*3M*) CRS-R <7 subgroup | .058 | .844 |
| CRS-R arousal change (*3M–6M*) to daily ITB dose (*6M*) CRS-R <7 subgroup | .035 | .907 |
|  |  |  |
| CRS-R arousal change (*PRE–3M*) to daily ITB dose (*3M*) CRS-R >7 subgroup | .171 | .594 |
| CRS-R arousal change (*3M–6M*) to daily ITB dose (*6M*) CRS-R >7 subgroup | --- | --- |
|  |  |  |
|  |  |  |
